# Clinical Safety and Tolerability of *Bifidobacterium bifidum* BBi32: An 8‐Week Randomized, Double‐Blind, Placebo‐Controlled Trial With Genomic and In Vitro Corroboration

**DOI:** 10.1002/fsn3.71420

**Published:** 2026-01-09

**Authors:** Shuguang Fang, Shanni Wang, Yinhua Liu, Chengsheng Zhu, Sijia Wang, Fei Xu

**Affiliations:** ^1^ Wecare Probiotics R&D Centers (WPC) Wecare Probiotics Co., Ltd. Suzhou China; ^2^ College of Biology Henan University of Technology Zhengzhou China; ^3^ College of Food Science and Technology Henan University of Technology Zhengzhou China

**Keywords:** *Bifidobacterium bifidum*, clinical trial, probiotic, safety assessment, toxicology

## Abstract

*Bifidobacterium bifidum* BBi32, isolated from a healthy infant, underwent a multi‐tiered safety assessment to evaluate its genetic features, in vitro properties, and effects on gut microbiota and host biomarkers. Whole‐genome sequencing (WGS) and functional annotation were performed alongside in vitro assays assessing acid and bile tolerance, mucin degradation, hemolysis, Caco‐2 cytotoxicity, and antibiotic susceptibility. Acute oral toxicity was tested in mice. A randomized, double‐blind, placebo‐controlled clinical trial (*n* = 40, 8 weeks) evaluated tolerability and exploratory endpoints, including hematology, liver and renal function, LL‐37 levels, gastrointestinal symptom scores, and 16S rRNA‐based microbiome profiling. The BBi32 genome comprised a 2.2 Mbp circular chromosome with 99.99% average nucleotide identity to the type strain, no plasmids, and no acquired antimicrobial resistance or virulence genes. Functional categories were enriched for ABC transporters, purine metabolism, and defense mechanisms. BBi32 demonstrated tolerance to acid and bile, lacked mucin‐degrading, or hemolytic activity, showed no cytotoxicity to Caco‐2 cells, and was susceptible to most antibiotics. Acute toxicity test yielded an LD_50_ > 2 × 10^10^ CFU/kg with no adverse effects. In the clinical trial, daily BBi32 administration (3 × 10^10^ CFU) was well tolerated, with no hematological or hepatic abnormalities. Compared with placebo, BBi32 reduced uric acid, urea, and creatinine levels, increased LL‐37, and improved gastrointestinal symptom scores. Microbiome analysis revealed higher alpha diversity, distinct community clustering, enrichment of *Romboutsia*, and predicted functional shifts toward amino acid biosynthesis and peptidase activity. Genomic, in vitro, toxicological, and clinical data collectively indicate that BBi32 meets key safety criteria and favorably modulates host and microbiome biomarkers, supporting its probiotic potential.

## Graphical abstract

An 8‐week randomized, double‐blind, placebo‐controlled trial found BBi32 well tolerated with no hematologic or hepatic abnormalities. Versus placebo, BBi32 reduced uric acid/urea/creatinine, increased LL‐37, and lowered gastrointestinal symptom scores. 16S profiling showed higher alpha diversity, distinct clustering, and Romboutsia enrichment—supporting safety and a microbiome‐mediated benefit on metabolic and mucosal‐immune biomarkers.

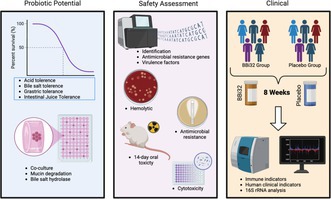

## 1 Introduction

Microorganisms include bacteria, fungi, viruses, and protozoa which are widespread in nature (Pepper and Gentry 2015). They are distributed in soil, water, air, and within living organisms (Szutowska 2020). Among them, probiotics constitute a group of beneficial microorganisms that, when administered in adequate amounts, confer health benefits to the host, as defined by the World Health Organization (WHO) (Hill et al. 2014; Vinderola et al. 2019). The human gut is a complex micro‐ecosystem, in which microorganisms and humans maintain a close and stable relationship (Sekirov et al. 2010). These microbes play essential roles in digestion, nutrient metabolism, and immune modulation, thereby supporting overall human health and attracting considerable scientific interest (Sekirov et al. 2010).

*Bifidobacterium* is among the most extensively studied and widely utilized probiotic genera. Within this genus, *Bifidobacterium bifidum* has garnered particular attention for its potential benefits in promoting gut health and modulating immune responses (Hidalgo‐Cantabrana et al. 2017). *B. bifidum* is a Gram‐positive, anaerobic bacterium naturally present in the human gastrointestinal tract, particularly abundant in healthy infants. It is commonly incorporated into probiotic supplements and functional foods due to its ability to maintain gut microbiota balance and support overall health (Hidalgo‐Cantabrana et al. 2017; O'Callaghan and van Sinderen 2016).

*Bifidobacterium bifidum* BBi32 was isolated from the feces of a healthy infant in Sichuan, China. BBi32 is manufactured by Wecare Probiotics Co. Ltd. as a stable, milky‐white or light‐yellow freeze‐dried powder. Owing to its stability and functionality, it is widely used in foods, dietary supplements, personal care products, pharmaceuticals, and agricultural applications.

**FIGURE 1:**
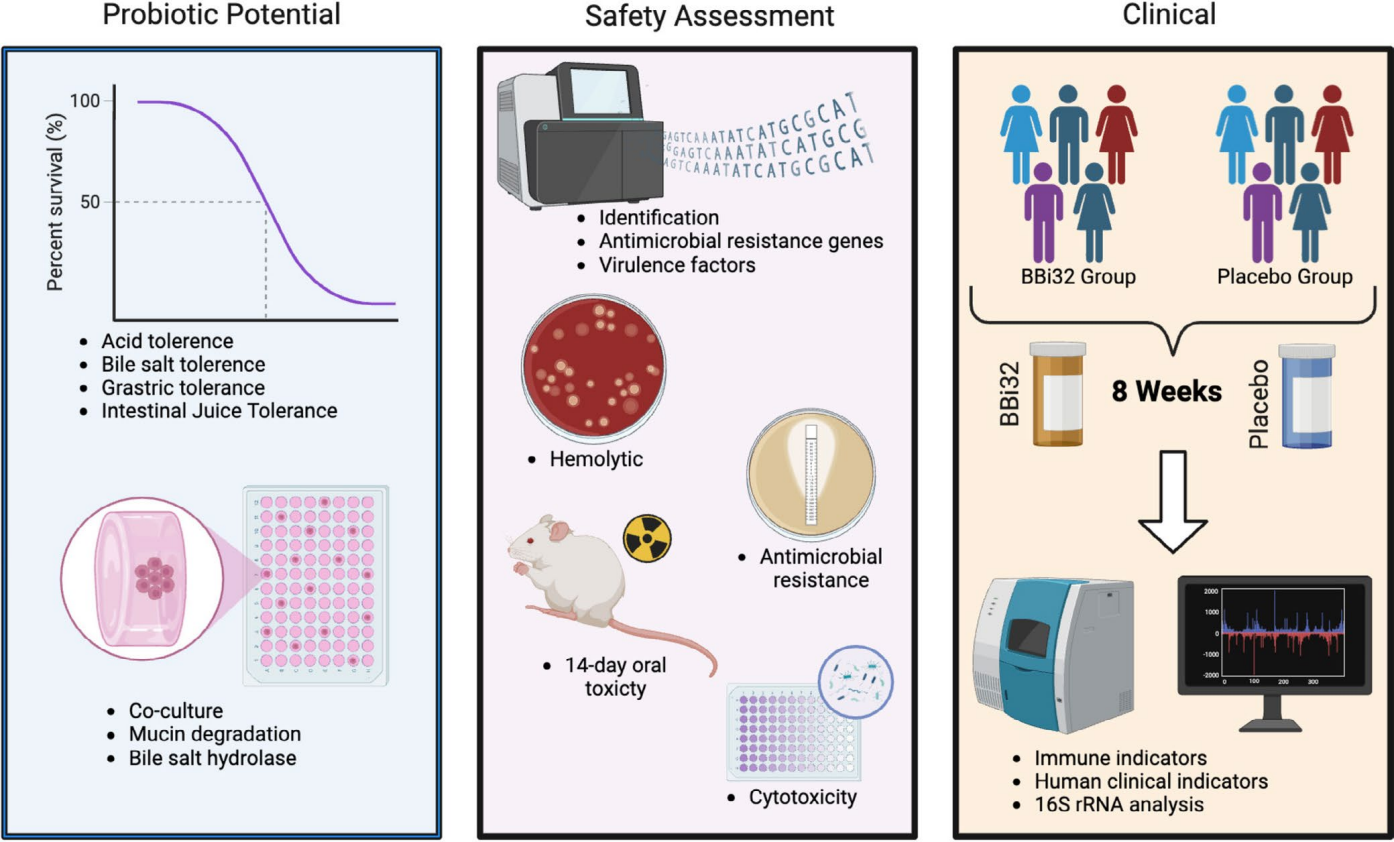
Graphical abstract.

This study aims to integrate WGS, resistance gene and toxigenic gene analysis, KEGG/GO/COG functional annotation, and acute toxicity test to systematically uncover the genetic features and environmental adaptation mechanisms of target pathogens. By analyzing the genomic structure of the strains based on WGS data, we focus on the distribution of resistance genes and virulence factors, and utilize KEGG metabolic pathways, GO biological processes, and COG functional categories to elucidate potential safety and functionality of BBi32 (Lv et al. 2017; Chen et al. 2024; Satti et al. 2018). Furthermore, acute toxicity assays will be performed to verify potential safety risks. Together with an 8‐week randomized, double‐blind clinical trial administering 3 × 10^10^ CFU/day to confirm the safety of BBi32 in humans (Figure 1). The primary research hypothesis is that BBi32 is genetically and toxicologically safe for human consumption, and on this basis, it is further hypothesized that BBi32 may exert beneficial effects on gut health and immune‐related functions in humans.

## 2 Materials and Methods

### 2.1 Strain Source and Culture

The strain *Bifidobacterium bifidum* BBi32 was originally isolated from the feces of a healthy infant in Sichuan, China, by Wecare Probiotics Co. Ltd. The strain has been deposited in the China General Microbiological Culture Collection Center (CGMCC) under the accession number CGMCC 16923 and in the Leibniz Institute DSMZ under the accession number DSM 34758. Cells were cultured anaerobically in MRS medium at 37°C for 18–24 h and harvested by centrifugation at 8000 ***g*** for 5 min (Li et al. 2024).

### 2.2 Genomic Sequencing

For genome sequencing, whole genome was extracted from BBi32 bacteria powder using the Dneasy Blood & Tissue Kit (Qiagen, batch number: 160028881), and quantified with a Qubit fluorometer (Invitrogen, batch number: 1948598) (Hull et al. 2018). Using the standard Illumina TruSeq Nano DNA LT library preparation protocol and the standard PacBio Template Prep Kit 1.0 were used to acquire the standard library separately (Zhao et al. 2021). After the quality control of the raw data by fastp (Chen et al. 2018), the clean data were assembled using Unicycler and polished to produce a complete circular genome (Wick et al. 2017).

### 2.3 Analysis of Risk Genes and Gene Function Classification

Species classification was determined using ANIclustermap (Jain et al. 2018). Antimicrobial resistance (AMR) genes were annotated through a comprehensive analysis utilizing AMRFinderPlus, the Comprehensive Antibiotic Resistance Database (CARD), and the ResFinder database (Feldgarden et al. 2019; McArthur et al. 2013; Bortolaia et al. 2020). Virulence genes were identified using VirulenceFinder and the Virulence Factor Database (VFDB) (Malberg Tetzschner et al. 2020; Liu et al. 2019). Pathogenic potential was evaluated via PathogenFinder (Cosentino et al. 2013). Additionally, genes associated with biogenic amine biosynthesis were identified through homology searches against the Kyoto Encyclopedia of Genes and Genomes (KEGG) and the Non‐Redundant Protein (NR) databases (Moriya et al. 2007; Camacho et al. 2009).

Metabolic product‐related genes were annotated using the KEGG Automatic Annotation Server (KAAS), mapping protein‐coding genes to KEGG Orthology (KO) and pathways (Moriya et al. 2007). GO and COG annotations were both performed using eggNOG‐mapper, which assigned genes to Gene Ontology (GO) categories (biological processes, molecular functions, and cellular components) and Clusters of Orthologous Groups (COG) functional classifications (Ashburner et al. 2000; Tatusov et al. 2003). Using the R (Version: 4.0.1) and ggplot2 to plot the corresponding figures (Gustavsson et al. 2022).

### 2.4 In Vitro Physiological and Biochemical Assays

Tolerance of strain BBi32 was evaluated by exposing cells to simulated gastrointestinal conditions, including acidic, gastric and intestinal fluids, and bile salts (Charteris et al. 1998; Dunne et al. 2001; Dunne 2001). BBi32 suspensions were inoculated into MRS broth adjusted to pH 2.5 and 3.0 using HCl, followed by anaerobic incubation at 37°C for 2 h. After incubation, cell viability was determined by colony counting on MRS agar. Simulated gastric juice was prepared by adding 0.5% NaCl and pepsin, and its pH was adjusted to 2.0–3.0. The suspensions were incubated anaerobically at 37°C, and viable counts were assessed. Simulated intestinal juice was prepared using pancreatin at pH 8.0, and viability was determined under the same conditions. For bile salt tolerance, BBi32 cells were cultured in MRS broth supplemented with 0.1% (w/v) porcine bile salts and incubated anaerobically at 37°C for 2 h prior to CFU determination.

To assess mucosal interaction potential, mucin degradation was evaluated. BBi32 was cultured in CHL medium containing 0.3% mucin for 48 h. Supernatants were analyzed using SDS‐PAGE followed by Coomassie Brilliant Blue staining to detect mucin degradation (Ruiz et al. 2013; Macfarlane and Macfarlane 2011).

Bile salt hydrolase (BSH) activity was evaluated by inoculating BBi32 onto MRS agar containing 0.5% sodium deoxycholate, 0.2% sodium thioglycolate, and 0.2% CaCl_2_. Following 72 h of anaerobic incubation at 37°C, transparent halos around colonies indicated BSH activity (Grill et al. 1995). Hemolytic activity was assessed on 5% sheep blood agar incubated at 37°C for 24 h to observe β‐hemolysis zones (Ammor et al. 2007). D‐/L‐lactate concentrations were quantified using D‐/L‐Lactic Acid Kits (r‐biopharm, Germany); absorbance values (A1 and A2) were measured at 37°C and used to calculate D‐lactate and total lactate according to the manufacturer's instructions.

Cytotoxicity was assessed by seeding Caco‐2 cells (1.5 × 10^4^ cells/well) into 96‐well plates and exposing them to 10% and 20% BBi32 fermentation supernatants for 24 h. Cell viability was measured using the CCK‐8 assay by detecting absorbance at 450 nm. For live bacterial cytotoxicity, Caco‐2 cells were co‐incubated with different concentrations of BBi32 (10^7^, 10^8^, and 10^9^ CFU/mL) suspensions for 4 or 8 h (Akbari et al. 2017; Cao et al. 2019). Lactate dehydrogenase (LDH) release was measured using the Promega LDH‐Glo Cytotoxicity Kit to evaluate membrane integrity and cytotoxic potential. Antibiotic susceptibility was determined using the broth microdilution method in accordance with EFSA and ISO guidelines. BBi32 suspensions (3 × 10^5^ CFU/mL) were added to 96‐well plates containing serial dilutions of 15 commonly used antibiotics, including aminoglycosides (e.g., streptomycin, gentamicin), macrolides (e.g., erythromycin), tetracyclines, chloramphenicol, and β‐lactams (e.g., ampicillin) (Gueimonde et al. 2013). Plates were incubated anaerobically at 37°C for 48 h, and minimum inhibitory concentrations (MICs) were recorded. Results were interpreted based on EFSA microbiological cut‐off values, and *Lacticaseibacillus paracasei* ATCC 334 was used as the quality control strain.

### 2.5 The 14‐Day Oral Toxicity Test

A single‐dose 14‐day acute oral toxicity study was conducted under Specific Pathogen Free (SPF) condition (temperature 20°C–22°C, humidity 45%–65%, 12‐h light–dark cycle), using 20 healthy ICR mice (10 males, 10 females, from Shanghai Shengchang Biological Technology Co. Ltd.), weighing 18–22 g (NIH 2011; Walum 1998). The mice were housed in plastic box units with corncob bedding and provided with sterilized drinking water and contaminant‐free feed. BBi32, with a content of 1 × 10^11^ CFU/g, was dissolved in sterile deionized water and administered via gavage at the maximum feasible dose (limit dose) of 2000 mg/kg body weight (0.2 mL per 10 g body weight). Clinical observations were performed immediately after dosing and twice daily thereafter. Prespecified endpoints included general appearance and activity, respiration, secretions (salivation/lacrimation), detailed assessment of fur/skin condition (e.g., piloerection), behavioral and CNS effects (e.g., gait, posture, tremors, convulsions, reactivity to handling), feces/urine consistency, food and water intake, body weights (days 0, 7, and 14), and mortality (Clements et al. 2022). All experimental procedures involving animals were reviewed and approved by the Animal Ethics Committee of the Shanghai Customs Center for Inspection and Quarantine of Animals, Plants, and Food (Approval No. 232024004633).

### 2.6 Clinical Trails

This clinical trial, conducted in Henan Province, China, from January to May 2025, adhered to the Declaration of Helsinki. It was approved by the Ethics Committee of Henan University of Technology. Written informed consent was obtained from all participants, ensuring voluntary participation with a clear understanding of objectives, procedures, and risks. The trial was registered at ClinicalTrials.gov (NCT06886711) before enrollment completion.

Healthy adults aged 18–45 were recruited. Inclusion criteria included: ability to follow study procedures, normal/corrected vision and hearing, agreement to daily probiotic/placebo intake, attendance at three on‐site follow‐ups, and provision of blood, urine, and fecal samples (two sets each) at baseline and intervention end. Exclusion criteria included: gastrointestinal diseases (e.g., celiac, ulcerative colitis, Crohn's), severe neurological disorders (e.g., epilepsy, stroke), psychiatric conditions or current treatment for alcohol/drug dependence, psychosis, schizophrenia, or bipolar disorder, antidepressant/mood‐stabilizer use, major organ failure (heart, liver, kidney), radiotherapy/chemotherapy history, general anesthesia surgery in the past 3 years or planned within 3 months, and infections (hepatitis B/C, HIV, syphilis). Recruitment occurred via online platforms and ads; eligible volunteers were enrolled by coordinators, assigned numbers, and randomly allocated to groups.

Participants received unique identifiers and were assigned to the BBi32 (30 billion CFU/3 g freeze‐dried powder per sachet/day) or Placebo (dextrin) group, with identical appearance and packaging managed by a third party. Blinding extended to participants, investigators, physicians, sample handlers, and analysts, with sealed code envelopes held by a third party for emergencies. Unblinding occurred in two stages post‐data collection by an independent party. Participants maintained daily intake for 8 weeks, avoiding additional probiotics, antibiotics, or gut supplements, with follow‐ups at baseline (BBi32_0 and Placebo_0) and Week 8 (BBi32_8 and Placebo_8) for samples and questionnaires.

Primary clinical observation indicators were baseline (Week 0) and Week 8, with assessments from 08:00 to 10:00 under fasting conditions (12+ hours), avoiding exercise and alcohol. Primary endpoints were LL‐37 (measured with Hycult Biotech ELISA kit, Catalog #HK321) and calprotectin (BÜHLMANN Quantum Blue immunoassay) levels. Secondary endpoints included: immunoglobulin analysis (IgA, IgG, IgM via Elabscience ELISA kits, Catalog #E‐EL‐H6002/H6001/H6003) and routine parameters (Sysmex XE‐5000, Dirui H‐500, Roche Cobas 8000 for blood biochemistry). The Gastrointestinal Symptom Rating Scale (GSRS), developed by Svedlund, is a 15‐item interview‐based scale used to assess gastrointestinal symptoms, with its reliability and validity confirmed in multiple studies, and was employed to statistically evaluate gut improvement before and after intervention (Svedlund et al. 1988). By collecting the feces of the subjects, gut microbiota profiling (16S rRNA V3–V4 with primers F1: 5′‐CCTACGGGNGGCWGCAG‐3′, R2: 5′‐GACTACHVGGGTATCTAATCC‐3′), sequenced on Illumina MiSeq, processed with QIIME2/DADA2 (Bolyen et al. 2019; Callahan et al. 2016), annotated against SILVA. Microbial diversity was assessed through α‐diversity indices (Shannon, Chao1) and β‐diversity metrics (Bray–Curtis, UniFrac), visualized via Principal Coordinates Analysis (PCoA). Differential taxa analysis was conducted using LEfSe (Linear Discriminant Analysis Effect Size), and functional predictions were inferred using PICRUSt2 (v2.5.0) (Segata et al. 2011; Douglas et al. 2020). Additional statistical comparisons for KEGG pathways were carried out with STAMP (v2.1.3) for group‐level significance testing (Parks et al. 2014).

For statistical analysis, hematology data were processed in R software (version 4.0.1). Two‐way ANOVA was applied to determine statistical significance. Data visualization for hematology and microbiome analysis was performed using ggplot2 within R.

## 3 Results

### 3.1 Genomic Analysis

**FIGURE 2:**
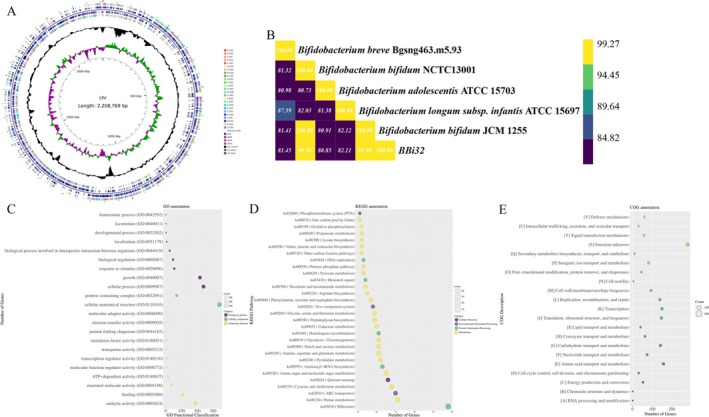
Whole‐genome sequencing and bioinformatic analysis of Bifidobacterium bifidum BBi32.

The genome of BBi32, obtained through sequencing, consists of a circular DNA molecule with a length of 2.2 Mbp and a GC content of 62.66% (Figure 2A). No plasmids were identified in BBi32 (Figure 2A). Based on Average Nucleotide Identity (ANI) analysis (Figure 2B), the genome size of BBi32 shows a 99.99% similarity to those of *Bifidobacterium bifidum* strains JCM 1255 and NCTC13001 (Figure 2B).

### 3.2 AMR and Virulence Genes

Analyses using AMRFinder and ResFinder identified no homologous antimicrobial resistance (AMR) genes in BBi32. However, the CARD database annotated an antimicrobial resistant gene—the 30S ribosomal protein S12 (rpsL) gene, located at positions 1,518,658–1,518,966 bp in the BBi32 genome. Virulence gene annotation, compared against VirulenceFinder and VFDB, showed no homologous virulence proteins in BBi32. Additionally, PathogenFinder estimated a low probability (0.246) of BBi32 acting as a human pathogen, thereby classifying it as non‐pathogenic. No biogenic amine‐related genes were detected in the BBi32 genome.

### 3.3 Gene Function Annotation

Functional annotation via Gene Ontology (GO) revealed that BBi32 genes are primarily involved in catalytic activity, protein‐containing complexes, cellular anatomical structures, cellular processes, and growth (Figure 2C). Pathway enrichment analysis highlighted significant involvement in ABC transporters, purine metabolism, and ribosome‐related pathways (Figure 2D). Annotation of homologous protein clusters showed that, aside from over 200 proteins with unknown functions, the majority are associated with amino acid transport and metabolism, carbohydrate transport and metabolism, replication, recombination, and repair, as well as transcription—functions critical for bacterial survival (Figure 2E). Notably, BBi32 contains a distinct group of homologous proteins dedicated to defense mechanisms (Figure 2E).

### 3.4 In Vitro Tolerance Tests

**FIGURE 3:**
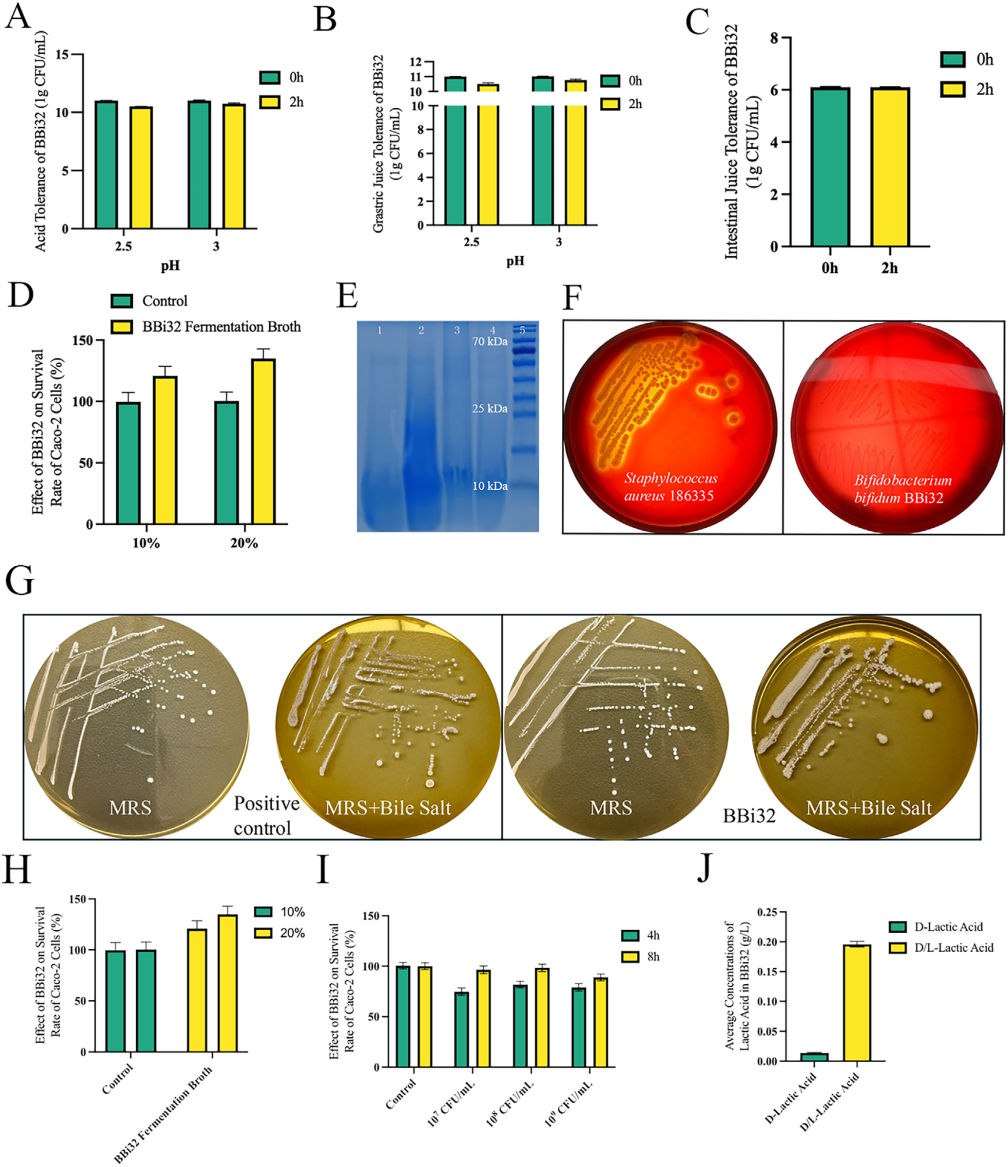
Acid tolerance and bile salt tolerance analysis of Bifidobacterium bifidum BBi32.

BBi32 retained 5.94% survival after 2 h in MRS medium at pH 2.5 and 11.94% at pH 3.0 (Figure 3A). In simulated gastric fluid, survival reached 84.05% at pH 2.5 and 97.51% at pH 3.0 after 2 h (Figure 3B). In simulated intestinal fluid, the 2‐h survival rate was 99.97% (Figure 3C). Exposure to 0.1% porcine bile salts yielded a 2‐h survival rate of 38.43% (Figure 3D). SDS‐PAGE analysis of mucin proteins showed no observable degradation, confirming BBi32's inability to degrade mucin (Figure 3E). BBi32 also exhibited no bile salt hydrolase activity or hemolytic properties (Figure 3F,G) and produced minimal D‐lactic acid and D/L‐lactic acid (Figure 3J).

### 3.5 Cytotoxicity

Cytotoxicity evaluations revealed that BBi32 fermentation supernatant (at 10% and 20% concentrations) supported robust Caco‐2 cell growth after 24 h of co‐incubation, achieving 120.19% and 133.92% of initial cell density, respectively (Figure 3H). Co‐incubation with BBi32 cells at concentrations of 10^7^, 10^8^, and 10^9^ CFU/mL for 4 or 8 h maintained Caco‐2 survival rates above 88% (Figure 3I).

### 3.6 Antibiotic Minimum Inhibitory Concentration

According to the EFSA guidelines, BBi32's in vitro minimum inhibitory concentrations (MICs) for antibiotics confirmed sensitivity across eight classes compared to the reference strain: ampicillin (0.064 μg/mL), vancomycin (0.032 μg/mL), gentamicin (16 μg/mL), streptomycin (16 μg/mL), erythromycin (0.032 μg/mL), clindamycin (0.032 μg/mL), tetracycline (2 μg/mL), and chloramphenicol (1 μg/mL) (Table 1).

**TABLE 1:** The minimum inhibitory concentration (MIC) of various antibiotics.

Group	1	2	3	4	5	6	7	8	9	10	11	12	13	14
Ampicillin	16−	8−	4−	2−	1−	0.5−	0.25−	0.125−	0.064−	0.032+	0.016+	0.008+	0.004+	N−
Vancomycin	4−	2−	1−	0.5−	0.25−	0.125−	0.064−	0.032−	0.016+	0.008+	0.004+	0.002+	0.001+	N−
Gentamicin	256−	128−	64−	32−	16−	8+	4+	2+	1+	0.5+	0.25+	0.125+	0.0625+	N−
Streptomycin	32−	16−	8+	4+	2+	1+	0.5+	0.25+	0.125+	0.064+	0.032+	0.016+	0.008+	N−
Erythromycin	8−	4−	2−	1−	0.5−	0.25−	0.125−	0.064−	0.032−	0.016+	0.008+	0.004+	0.002+	N−
Clindamycin	16−	8−	4−	2−	1−	0.5−	0.25−	0.125−	0.064−	0.032−	0.016+	0.008+	0.004+	N−
Tetracycline	2−	1+	0.5+	0.25+	0.125+	0.064+	0.032+	0.016+	0.008+	0.004+	0.002+	0.001+	0.0005+	N−
Chloramphenicol	8−	4−	2−	1−	0.5+	0.25+	0.125+	0.064+	0.032+	0.016+	0.008+	0.004+	0.002+	N−

### 3.7 The 14‐Day Oral Toxicity Test

No mortality occurred during the 14‐day observation period in ICR mice following single‐dose oral gavage of BBi32 (at the dose of 2 × 10^10^ CFU/kg). Across prespecified endpoints (appearance/activity, respiration, secretions, fur/skin, feces/urine), no treatment‐related clinical signs were observed. Food and water intake and body‐weight gain were comparable to the negative control in both females and males (*p* > 0.05, Table 2). At termination, terminal gross (macroscopic) examination showed no treatment‐related lesions. Collectively, these results indicate that the oral LD_50_ exceeds the tested limit dose under the conditions of this study.

**TABLE 2:** Acute oral toxicity of BBi32.

Dose (CFU/kg)	Sex	No. of animals	Body weight (*x* ± SD, g)			Death animals	Mortality (%)
			0	7	14		
2 × 10^10^	Female	10	18.4 ± 0.52	24.2 ± 0.63	28.3 ± 0.67	0	0
	Male	10	18.2 ± 0.42	26.5 ± 0.85	34.4 ± 0.97	0	0

### 3.8 Clinical Assessment

**FIGURE 4:**
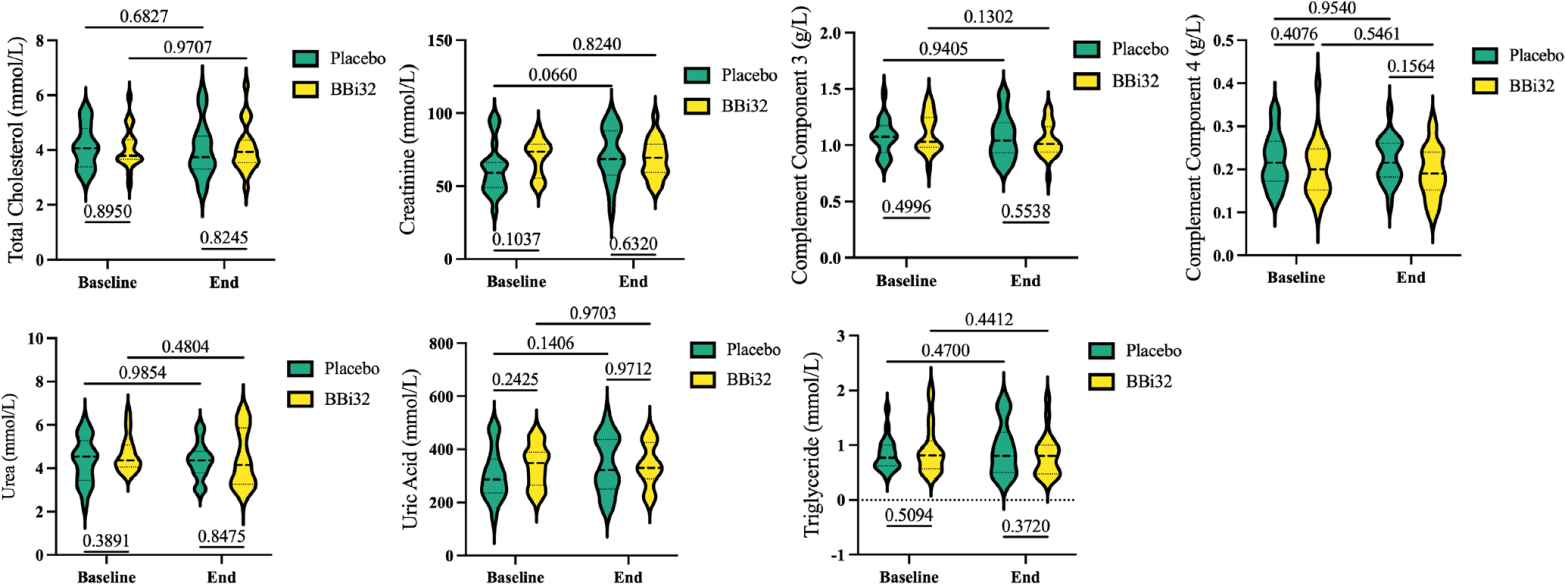
Serum biomarker analysis comparing Bifidobacterium bifidum BBi32 and placebo groups at baseline and end of treatment.

Following an 8‐week clinical trial, 40 participants completed the study (20 in the probiotic group and 20 in the placebo group). Routine blood analyses revealed no differences in parameters between the 8‐week probiotic group (BBi32_8) and the 8‐week placebo group (Placebo_8) (Table 3). Red blood cell count, white blood cell count, hemoglobin, hematocrit, platelet count, and lymphocytes showed no significant changes (*p* > 0.05) within or between groups. Liver enzymes (AST, ALT) also exhibited no inter‐group differences. Additional blood parameters showed no significant variations (*p* > 0.05) in total cholesterol, complement 3/4, or triglycerides. However, compared to BBi32_0, BBi32_8 displayed moderate reductions in uric acid, urea, and creatinine (Figure 4).

**TABLE 3:** Changes in serum biochemical indicators before and after intervention.

	Placebo (*n* = 20)	BBi32 (*n* = 20)	*p*
White Blood Cell Count (10^9^/L)			
Baseline	6.12 ± 1.39	5.84 ± 1.65	0.263
End	5.77 ± 1.20	6.08 ± 1.73	0.254
*p*‐value (intergroup)^2^	0.141	0.262	
Red Blood Cell Count (10^12^/L)			
Baseline	4.64 ± 0.65	4.78 ± 0.48	0.164
End	5.20 ± 0.76	4.84 ± 0.50	0.055
*p*‐value (intergroup)^2^	0.167	0.321	
Hemoglobin (g/L)			
Baseline	146.65 ± 16.40	144.35 ± 16.57	0.301
End	152.00 ± 15.83	147.80 ± 18.22	0.220
*p*‐value (intergroup)^2^	0.069	0.222	
Hematocrit (%)			
Baseline	43.79 ± 5.37	43.99 ± 4.96	0.885
End	45.14 ± 4.78	43.89 ± 5.21	0.638
*p*‐value (intergroup)^2^	0.33	0.181	
Platelet Count (10^9^/L)			
Baseline	265.15 ± 50.39	252.85 ± 44.00	0.431
End	258.65 ± 52.26	258.05 ± 41.77	0.227
*p*‐value (intergroup)^2^	0.168	0.467	
Alanine Aminotransferase (U/L)			
Baseline	23.90 ± 21.35	16.24 ± 11.05	0.070
End	18.80 ± 5.33	18.90 ± 8.58	0.480
*p*‐value (intergroup)^2^	0.152	0.101	
Aspartate Aminotransferase (U/L)			
Baseline	21.30 ± 9.48	15.64 ± 5.53	0.098
End	19.45 ± 3.32	17.75 ± 3.32	0.068
*p*‐value (intergroup)^2^	0.149	0.060	
Granulocytes (10^9^/L)			
Baseline	52.69 ± 7.60	49.36 ± 8.91	0.162
End	49.22 ± 10.23	49.18 ± 8.34	0.495
*p*‐value (intergroup)^2^	0.146	0.475	
Lymphocytes (10^9^/L)			
Baseline	2.27 ± 0.57	2.27 ± 0.55	0.481
End	2.47 ± 0.73	2.40 ± 0.70	0.163
*p*‐value (intergroup)^2^	0.159	0.205	

### 3.9 Immunity Index

**FIGURE 5:**
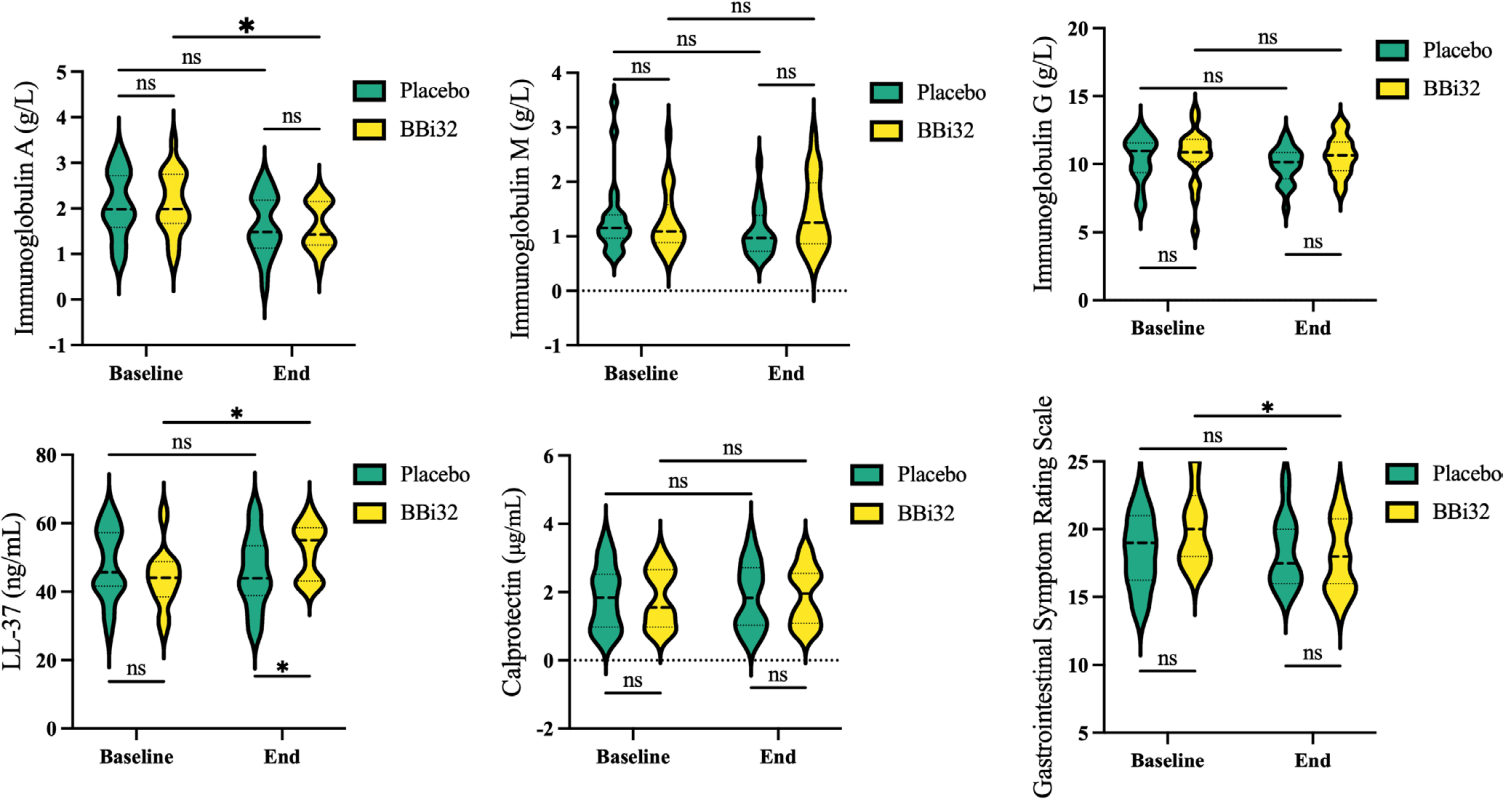
Evaluation of immune‐related indicators and Gastrointestinal Symptom Rating Scale in Bifidobacterium bifidum BBi32 and placebo groups.

Figure 4 illustrates that, compared to Placebo_8, BBi32_8 exhibited elevated levels of immunoglobulin A (IgA), immunoglobulin M (IgM), immunoglobulin G (IgG), and calprotectin. Particularly, LL‐37 levels were significantly higher in BBi32_8 versus Placebo_8 (Figure 5). After 8 weeks of probiotic supplementation, Gastrointestinal Symptom Rating Scale scores reflected general improvements in intestinal health relative to baseline, with statistically significant differences between BBi32_8 and BBi32_0 (Figure 5).

### 3.10 16s Sequence Analysis

**FIGURE 6:**
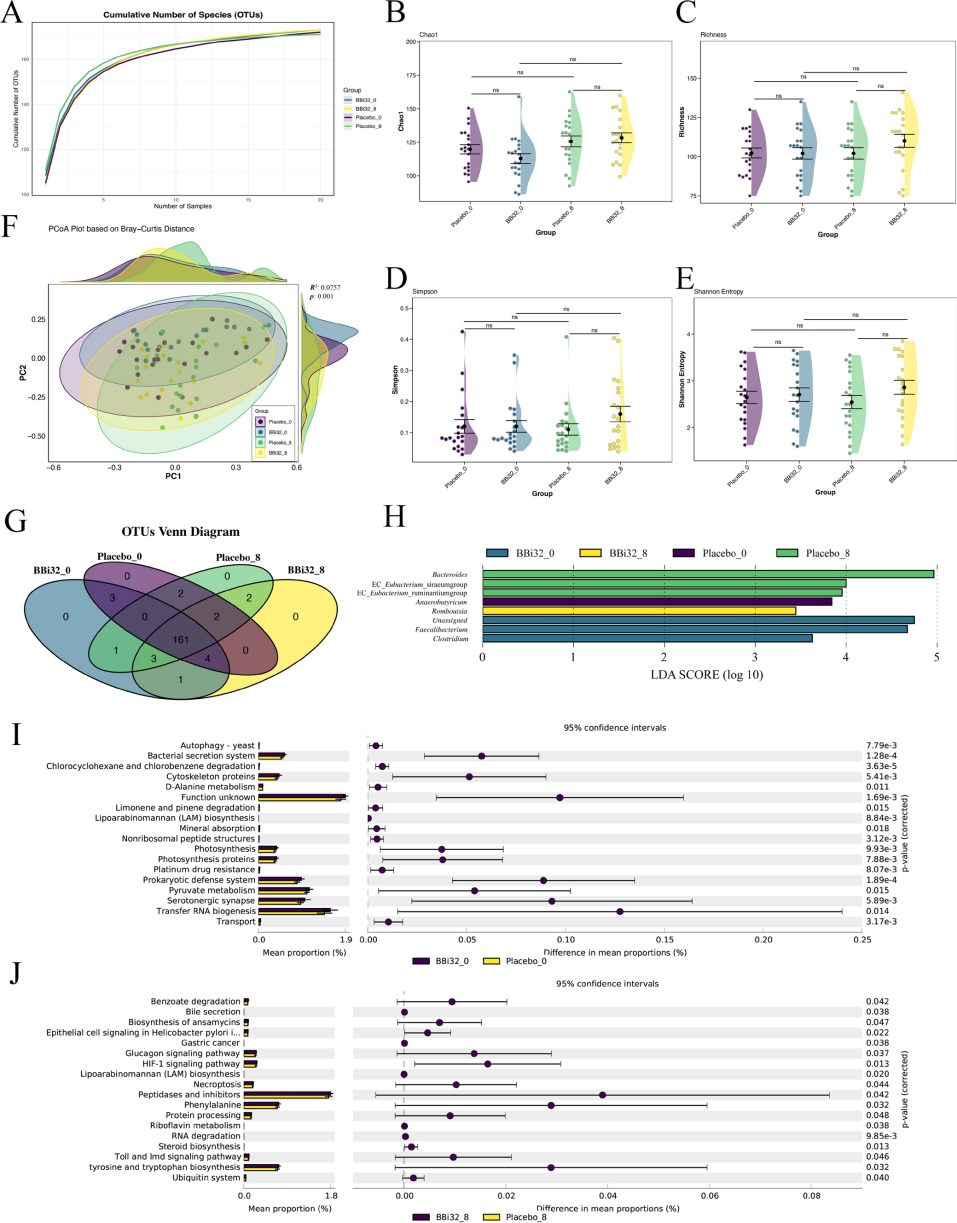
Microbial diversity and functional analysis of gut microbiota in response to Bifidobacterium bifidum BBi32 treatment.

After 8‐week intervention, operational taxonomic unit (OTU) rarefaction curves for all groups trended upward, with BBi32_8 displaying the highest cumulative OTU count (Figure 6A). Alpha diversity metrics (Chao1, Simpson, Shannon, and richness) indicated greater species abundance in BBi32_8 compared to Placebo_8 (Figure 6B–E).

This pattern held for BBi32_8 versus BBi32_0, underscoring increased microbial richness post‐probiotic intervention. Principal coordinates analysis (PCoA) using Bray‐Curtis distances revealed distinct clustering for BBi32_8, confirming that probiotic supplementation reshaped the overall gut microbiota structure (Figure 6F). PERMANOVA analysis confirmed significant differences in microbial community composition (*R* ^2^ = 0.076, *p* = 0.001), with treatment and timepoint explaining 7.6% of the total variation in beta diversity. Venn diagram analysis identified 161 shared OTUs across probiotic and placebo groups pre‐ and post‐intervention, with BBi32_8 harboring 4 unique OTUs relative to BBi32_0 (Figure 6G).

Linear discriminant analysis effect size (LEfSe) was performed across all four groups (BBi32_0, BBi32_8, Placebo_0, and Placebo_8) to identify group‐specific microbial biomarkers. Linear discriminant analysis effect size (LEfSe) highlighted group‐specific marker genera: *Romboutsia* in BBi32_8; *Faecalibacterium*, *Clostridium*, and unclassified genera at baseline for the probiotic group; *Bacteroides* in Placebo_8; and *Eubacterium*‐related genera plus *Anaerobutyricum* at baseline for the placebo group. All differential genera achieved linear discriminant analysis (LDA) scores > 3.0, signifying substantial inter‐group microbiota distinctions (Figure 6H).

STAMP analysis of functional genes revealed baseline differences (BBi32_0 vs. Placebo_0) in pathways such as transport, tRNA biosynthesis, serotonergic synapse, pyruvate metabolism, prokaryotic defense systems, and others (0.05%–0.25% variance; all *p* < 0.05) (Figure 6I). Post‐intervention (BBi32_8 vs. Placebo_8), shifts focused on ubiquitin systems, tyrosine and tryptophan biosynthesis, Toll and Imd signaling, steroid biosynthesis, RNA degradation, riboflavin metabolism, and other additional pathways (0.02%–0.08% variance; all *p* < 0.05) (Figure 6J). Pathways like benzoate degradation, ansamycin biosynthesis, phenylalanine metabolism, peptidases and inhibitors, tyrosine and tryptophan biosynthesis, and HIF‐1 signaling showed enriched gene annotations in BBi32_8 versus Placebo_8, with tyrosine and tryptophan biosynthesis, phenylalanine metabolism, and peptidases/inhibitors ranking as the top three by gene count, suggesting pivotal roles (Figure 6J).

## 4 Discussion

The probiotic potential of *Bifidobacterium bifidum* BBi32 hinges on its demonstrated safety profile and capacity to modulate gut microbiota, as evidenced by comprehensive genomic, in vitro, toxicological, and clinical evaluations. Genomic sequencing confirmed BBi32 circular chromosome without plasmids, aligning with non‐pathogenic traits observed in related strains, such as *Bifidobacterium bifidum* BGN4, where whole‐genome analysis similarly revealed no transferable AMR or virulence factors (Kim et al. 2018). The absence of homologous AMR genes via AMRFinder and ResFinder, coupled with only a single *rpsL* annotation in CARD (a ribosomal mutation often intrinsic and non‐transferable), underscores low risk for antibiotic resistance dissemination (Elnar et al. 2025). This is consistent with studies on *Bifidobacterium bifidum* IDCC strains, where genomic toxicity assessments confirmed safety through minimal AMR and virulence gene presence. Virulence assessments against VFDB and VirulenceFinder yielded no matches, and PathogenFinder's 0.246 pathogenicity probability further classifies BBi32 as safe, mirroring evaluations of *Bifidobacterium breve* BS2‐PB3, which lacked virulence factors and supported probiotic candidacy (Marbun et al. 2024).

BBi32's in vitro tolerance to low pH and bile salts exceeds thresholds for probiotic viability, enabling gastrointestinal transit (Kujawska et al. 2024). These traits compare favorably to screened *Bifidobacterium* isolates from infants, where acid and bile tolerance ranged from 45% to 55% survival, highlighting BBi32's robustness (Li et al. 2025). Antibiotic susceptibility per EFSA standards (low MICs across eight classes) indicates intrinsic sensitivity without acquired resistance, akin to patterns in *Bifidobacterium bifidum* strains exposed to bile, where tolerance did not compromise antibiotic profiles (Charteris et al. 2000). Absence of mucin degradation, bile salt hydrolase, and hemolysis further bolsters safety, as these activities could disrupt gut barriers in pathogenic contexts (Peng et al. 2023). Cytotoxicity assays showed no harm to Caco‐2 cells (survival > 88%; growth promotion up to 133.92%), paralleling non‐toxic effects in *Bifidobacterium bifidum* BGN4 safety evaluations (Kim et al. 2018). The 14‐day rat toxicity study (LD50 > 2 × 10^10^ CFU/kg; no abnormalities) and 8‐week human trial (no blood parameter changes; reduced uric acid/urea/creatinine) affirm in vivo safety, consistent with clinical assessments of *Bifidobacterium breve* IDCC4401, where no adverse effects emerged (Choi et al. 2021).

Beyond safety, BBi32 modulates gut microbiota and immunity, as revealed by 16S rRNA sequencing and blood analyses. Post‐intervention, increased OTU counts, alpha diversity (Chao1/Shannon/Simpson/richness), and distinct PCA clustering in BBi32_8 indicate enhanced microbial richness and structural shifts, aligning with trials where *Bifidobacterium bifidum* supplementation altered gut composition in healthy adults, boosting beneficial taxa (Gargari et al. 2016). LEfSe‐identified markers like *Romboutsia* in BBi32_8 suggest probiotic‐driven enrichment of health‐promoting genera, similar to changes in constipated patients treated with *Bifidobacterium bifidum* , where microbiota diversity rose alongside symptom relief (Wang et al. 2022). In support of these microbiota‐level changes, functional genomic annotation (GO, KEGG, and COG) revealed metabolic features directly linked to BBi32's probiotic adaptability. Notably, enrichment of ABC transporters, purine metabolism, and carbohydrate utilization pathways provides a mechanistic basis for its acid and bile tolerance, nutrient acquisition, and stress resilience. ABC transporters enable active efflux of bile salts, antimicrobial peptides, and toxic metabolites, thereby enhancing survival under gastrointestinal stress (Pfeiler and Klaenhammer 2009; Zhu et al. 2019). Meanwhile, purine metabolism contributes to ATP generation and DNA repair during acid or oxidative challenge, sustaining viability under harsh gut conditions. Enhanced carbohydrate metabolism and host‐glycan utilization suggest BBi32's competitive ability to colonize intestinal niches and support commensal stability (Bustos et al. 2025). STAMP functional analysis highlighted enriched pathways in BBi32_8, including tyrosine/tryptophan biosynthesis and peptidases (top gene counts), which may support amino acid metabolism and immune signaling, echoing metabolic shifts in *Bifidobacterium bifidum* PRL2010, involving ABC transporters and purine pathways for host‐glycan foraging (Turroni et al. 2010). These alterations correlate with improved Gastrointestinal Symptom Rating Scale scores, reduced uric acid/urea/creatinine, and elevated immunity indices (IgA/IgM/IgG/calprotectin/LL‐37), consistent with *Bifidobacterium* modulation of Treg‐dependent microbiota and IgA production in inflammatory models. Encapsulated *Bifidobacterium bifidum* has similarly potentiated intestinal IgA, while broader immune enhancements (e.g., IgG/IgM) align with probiotic effects in infection contexts (Park et al. 2002). Overall, BBi32's microbiota‐regulating role likely stems from its metabolic adaptations, such as purine/ABC transporter pathways, fostering symbiotic interactions that enhance barrier function and reduce inflammation (Hao et al. 2023). However, it is worth noting that the current study was conducted under free‐living conditions without a strictly standardized diet. While this reflects real‐world applicability, future investigations involving controlled dietary interventions are warranted to further isolate the specific contributions of BBi32 and validate these mechanistic insights.

## 5 Conclusion

In summary, *Bifidobacterium bifidum* BBi32 appears to be a safe probiotic candidate, with genomic, in vitro, and in vivo data suggesting the absence of AMR or virulence risks, good gastrointestinal tolerance, antibiotic sensitivity, and no observable toxicity in animal or human models. Its potential to enhance gut microbiota diversity, shift community structure toward beneficial genera, and support metabolic pathways such as amino acid biosynthesis indicates a possible role in intestinal regulation. Clinically, these changes were associated with trends toward improved immunity (LL‐37) and symptom relief, without apparent adverse effects on routine blood parameters. However, STAMP analysis revealed statistically significant differences in gut microbiota functional potential between the two groups at baseline (BBi32_0 vs. Placebo_0), suggesting a potential baseline imbalance in randomization. As a result, some of the between‐group differences observed post‐intervention cannot be unequivocally attributed to the BBi32 intervention, representing a key limitation of this study. This study had several limitations. First, the sample size was relatively small, consistent with the rule of thumb for pilot studies, and therefore the statistical power to detect subtle effects was limited. A larger cohort will be needed in future research to validate these preliminary findings. Second, no dietary assessments were incorporated, making it impossible to evaluate the potential influence of diet on gut microbiota composition and host metabolism. Moreover, several gut microbiota functional pathways differed significantly between the two groups at baseline, which limits our ability to attribute the post‐intervention changes solely to BBi32. Future studies should not only explore long‐term applications of BBi32 in specific clinical conditions but also systematically account for dietary factors and individual differences in the experimental population to more precisely delineate its role in gut health modulation, and it is also necessary to further investigate the association between BBi32 and its probiotic effects on immune modulation and gut health.

## Author Contributions

Conceptualization: Shuguang Fang and Shanni Wang; methodology: Shuguang Fang; software: Shuguang Fang; validation: Shuguang Fang and Chengsheng Zhu; formal analysis: Shuguang Fang; investigation: Shanni Wang; resources: Shuguang Fang; data curation: Shuguang Fang; writing – original draft preparation: Sijia Wang.; writing – review and editing: Yinhua Liu; visualization: Shuguang Fang; supervision: Yinhua Liu; project administration: Fei Xu. All authors have read and agreed to the published version of the manuscript.

## Conflicts of Interest

The authors declare no conflicts of interest.

## Data Availability

The data that support the findings of this study are available from the corresponding author upon reasonable request.
